# Editorial: Utilization of neuroscience core concepts to guide programs, curricula, courses, and assessment in diverse institutional contexts

**DOI:** 10.3389/fnint.2025.1595264

**Published:** 2025-04-01

**Authors:** Audrey Chen, Kimberley A. Phillips, Jennifer E. Schaefer, Patrick M. Sonner

**Affiliations:** ^1^Department of Neurobiology and Behavior, University of California, Irvine, Irvine, CA, United States; ^2^Department of Psychology, Trinity University, San Antonio, TX, United States; ^3^Department of Biology, College of Saint Benedict/Saint John's University, Collegeville, MN, United States; ^4^Department of Neuroscience, Cell Biology, and Physiology, Wright State University, Dayton, OH, United States

**Keywords:** core concepts, neuroscience, STEM higher education, curriculum, pedagogy

In 2023, we published a community-derived set of higher education neuroscience core concepts (Chen et al., 2023). Core concepts (CCs) are one type of educational framework that are comprised of overarching principles that frame knowledge. These are unique from other educational frameworks including core competencies and fundamental facts. Core competencies are skills that facilitate students becoming effective practitioners in a field and fundamental facts are basic information that is well understood but cannot explain phenomena across an array of contexts. The eight papers from the Research Topic “*Utilizing neuroscience core concepts to guide programs, curricula, courses, and assessment across diverse institutional contexts*” present ways in which neuroscience core concepts (NCC) have since been utilized, including: practical resources and suggestions for teaching (Shah et al.; Striedter; Cooper et al.; Hannah and Schaefer); developing majors and minors in neuroscience (Stocker and Duncan; Proksch et al.); evaluation of NCCs across neuroscience curricula (Maita et al.); and lessons learned in the development and use of CCs (Schaefer and Michael).

A number of articles describe the application of NCCs as a tool for identifying gaps and redundancies in courses across a program. Others use the NCCs as a tool for surveying their curriculum or to make decisions about which topics and ideas to prioritize within a program or specific courses. Maita et al. address how departments at one institution differ in the degree to which they actually incorporated and desired to incorporate NCCs into their neuroscience courses. Unsurprisingly, they noted differences in depth of coverage amongst individual psychology, cognitive science, and biology courses. Their in-depth analysis found that instructors would like to include more NCCs in their courses and identified, along with Stocker and Duncan, NCCs that were difficult to incorporate into specific courses, due to prioritizing other concepts based on focus of the course and instructor expertise. Their work provides other educators with ideas as to how they can assess their own courses/curricula to determine if some NCCs should be emphasized more or less.

While Maita et al. and Stocker and Duncan describe the incorporation of NCCs into existing neuroscience programs, Proksch et al. describe how NCCs were used to design a new interdisciplinary major curriculum at a small liberal arts college. They describe how the NCCs were mapped across introductory and advanced courses in a four-year program. While the NCCs described by Chen et al. (2023) encompass aspects of neuroscience related to humans and other species, Proksch et al. also chose to specifically highlight human applications in a separate learning outcome. Stocker and Duncan note an opportunity for how to engage both biology and psychology disciplinary strengths as instructors introduce the Gene-Environment Interaction concept into the neuroscience curriculum. Given that revisions to courses and curricula often require buy-in from various stakeholders, Schaefer and Michael provide communication strategies that may help educators realize the utility of CCs for such work.

A group of articles in the Research Topic also provides practical resources and suggestions for using NCCs to support teaching. Schaefer and Michael provide a set of tips that can assist educators in implementing disciplinary CCs in their own teaching. Cooper et al. examine novice students' understanding of the Evolution and Structure-Function Relationship NCCs at the outset of a general education neuroscience course for non-majors. Their findings–that a majority of non-biologists enter introductory courses with little comprehension of either concept and that preconceptions vary by student major–will help faculty predict areas of emphasis for applying NCCs in their courses and identify target areas for attending to common misconceptions. Striedter focuses specifically on the Evolution concept, making a strong case for why understanding nervous system evolution is essential for neuroscientists. Additionally, Striedter provides specific examples and ideas as to how educators can address this core concept in their teaching. Shah et al. provide resources and suggestions for navigating the rapidly-expanding, interdisciplinary field of neuroimmunology using an integrated core-concept based approach, providing a blueprint that may assist in course development or revision. Hannah and Schaefer describe an easily-replicated teaching activity that helps students learn to read primary literature using NCCs as a contextual framework. They provide data outlining the metacognitive processes identified by students as most benefiting from the use of NCCs to read primary literature. Finally, Stocker and Duncan suggest practical teaching activities that incorporate NCCs into coursework when faced with limited institutional resources, connecting classic published research papers, case studies, and 3-D printing of biomolecules to specific NCCs.

As the articles in this Research Topic indicate, there are a variety of benefits in using NCCs in neuroscience courses and curricula. However, such efforts by the neuroscience community are just beginning and will need to continue to adapt implementation of NCCs to the needs of students, courses, curricula, and institutions. An example of the continued work needed following development of NCCs can be seen in the work products that followed publication of Biology Core Concepts in *Vision and Change* (AAAS, 2011). Following the identification of the Biology Core Concepts, various instruments and frameworks for utilization by departments and programs were developed (Branchaw et al., 2020; Brownell et al., 2014; Cary et al., 2019). The work needed to effectively utilize NCCs has begun with publications in this Research Topic. We hope the articles in the Research Topic will provide neuroscience educators with ideas and inspiration for how they might utilize the NCCs in their courses, curricula, programs, and assessments and inspire continued application and research in this important field.

Looking toward the future, a key next step is unpacking the NCCs into the underlying conceptual elements within each core concept. We invite collaborators to join us in that work. Additionally, we envision that concept inventories and other assessment tools will be developed for the neuroscience education community to utilize. Finally, we are hopeful that the neuroscience community will continue the conversation about the most effective ways to utilize NCCs in neuroscience education.
